# Expression patterning reveals retinal inflammation as a minor factor in experimental retinopathy of ZDF rats

**DOI:** 10.1007/s00592-013-0550-2

**Published:** 2014-01-30

**Authors:** Paulus Wohlfart, Jihong Lin, Nadine Dietrich, Aimo Kannt, Ralf Elvert, Andreas W. Herling, Hans-Peter Hammes

**Affiliations:** 1 R&D Diabetes Division, Research and Translational Medicine, Sanofi, Industriepark Höchst, 65926 Frankfurt, Germany; 2 5th Medical Department, Universitätsmedizin Mannheim (UMM), University of Heidelberg, Mannheim, Germany

**Keywords:** Zucker diabetic fatty rats, Diabetic retinopathy, Preclinical model, Growth factors, Inflammation, Adhesion

## Abstract

Obese Zucker diabetic fatty (ZDF) rats are used as a type-2 diabetes model for microvascular complications. In order to study retinopathy in this model, changes in retinal vasculature were analyzed by quantitative morphometry and related to retinal expression of 46 selected genes that were analyzed by microfluidic card PCR technology. At 3 months of age, obese animals had developed stable hyperglycemia (20.7 ± 1.3 mmol/L plasma glucose vs. 6.5 ± 0.1 mmol/L in lean). Hyperinsulinemia initially presented in obese rats at 2 months (10.5 ± 0.7 μg/L plasma insulin vs. 0.2 ± 0.04 μg/L in lean) and decreased at 3 months (3.9 ± 0.6 vs. 0.5 ± 0.09 μg/ml in lean). At 8 months of age, animals had developed microvascular complications. An increased number of acellular capillaries in obese (24 ± 5/mm^2^) versus lean (15 ± 4/mm^2^) and a decreased number of retinal pericytes in obese (2,270 ± 250/mm^2^) versus lean animals (1,620 ± 243/mm^2^) could be observed. VEGFa, MIF, and HIF-1α were the most abundantly expressed and inflammatory genes such as TNFα and IL-6 are the least abundantly expressed genes. None of these genes were differentially regulated. Surprisingly, specific growth factors such as bFGF (FGF2) and placental growth factor, and adhesion molecules such as ICAM-1 were abundantly expressed and up-regulated in diabetic versus non-diabetic ZDF rats. In summary, we observed in type-2 diabetic ZDF rats retinopathy with retinal vasoregression along with a simultaneous up-regulation of specific growth factors such as bFGF and adhesion molecules, but only minor changes in key inflammatory genes.

**Electronic supplementary material:**

The online version of this article (doi:10.1007/s00592-013-0550-2) contains supplementary material, which is available to authorized users.

## Introduction

Diabetic retinopathy is a major complication of diabetes and observed in nearly all individuals with type-1 diabetes and in more than 80 % of individuals with type-2 diabetes after 20 years of disease. High glucose levels damage in particular the retina of diabetic patients and involve small vessel vasoregression and pericyte loss as initiating processes. However, although hyperglycemia is the most important single factor, it explains only partially the overall damage as indicated by results from large clinical trials [1].

Rodent animal models recapitulate incipient retinal damage, including vasoregression. However, most of the rodent models still build on chemical or genetically induced destruction of the pancreas, mimicking type-1 diabetes. Male obese Zucker diabetic fatty (ZDF) rats are a genetic model of type-2 diabetes, mostly used for the characterization of diabetic nephropathy, whereas retinopathy is described still incompletely. Danis and Yang demonstrated in retina of diabetic ZDF rats modestly increased numbers of cellular nuclei and an increased basement membrane thickness [2, 3]. An increased number of retinal acellular capillaries (AC) and apoptotic pericytes and endothelial cells were identified in aged diabetic ZDF rats and partially reduced by long-term treatment with a tumor necrosis alpha (TNFα) scavenger [4]. TNFα also mediated diabetes increased translocation of FOXO1 to nuclei in microvascular cells in vivo [5]. Up-regulation of retinal expression of TNFα, but also other markers of inflammation could be demonstrated in 14-week-old obese versus lean ZDF rats [6].

Inflammation may be an important disease mechanism in diabetes, and obese ZDF rats develop local and systemic inflammation with increased levels of circulating inflammatory biomarkers and increased infiltration of CD11b-positive cells into adipose tissue [7]. In a pilot study, we noticed, however, that in the retina, the expression of TNFα and other inflammatory markers falls clearly below other interesting factors and hence may be overestimated. We therefore investigated in male aged obese ZDF rats and lean littermates retinal morphology and, in parallel, associated gene expression pattern by a novel method of parallel quantitative PCR in microfluidic cards. This technology enables accurate assessment of abundance over a wide dynamic range and, in parallel, regulation of genes of interest. We selected genes of interest from published sources with an emphasis on growth factors, inflammatory markers, and adhesion molecules.

## Methods

Zucker diabetic fatty (ZDF-Lepr^fa^/Crl) rats were obtained from Charles River Laboratories (Brussels, Belgium) in two genotypes, obese ZDF (genotype Fa/Fa) and lean ZDF littermates (genotype Fa/?) and kept under standard housing. The principles of laboratory animal care (NIH publication No. 86-23, revised 1985) were followed, as well as the specific national law (the current version of the German Law on the Protection of Animals) and further Sanofi internal guidelines where applicable. Eight animals per group were used in all subsequent analyses. Diabetes was monitored weekly by blood glucose and insulin, and body weight measurements.

### Retinal preparation and quantification of morphometry

Retinal morphology was analyzed by retinal digest preparations and quantitative morphometry in 8-month-old ZDF rats as described [8]. Briefly, after fixation of eyes in 4 % PBS-buffered formaldehyde solution for 2 days, retinae were isolated and then incubated with trypsin. Following a periodic acid–Schiff (PAS)-hematoxylin staining, the number of total pericytes was evaluated in ten randomly selected fields per retina under 400× magnification using an image system and Cell^F^ analyzing software (Olympus, Hamburg, Germany). The cell numbers were normalized to relative capillary density (cell numbers/mm^2^ capillary area). AC were counted under 40× objective with a sample of PAS-stained retinal vasculature and normalized to relative retinal area (AC numbers/mm^2^ retinal area).

### Gene expression measurements

Expression of several genes was quantified in parallel by using TaqMan microfluidic card technology on an Applied Biosystems ViiA7 cycler (software version 1.1). Retinal RNA was isolated from individual retinas homogenized in 1 ml Trizol reagent (Invitrogen, Karlsruhe, Germany) according to the manufacturer’s instructions. Crude RNA was repurified using a RNeasy Mini Kit (Qiagen, Hilden, Germany) followed by reverse transcription into cDNA using a high-capacity kit (Life technologies, Darmstadt, Germany). Samples were mixed with TaqMan Universal PCR Master Mix (Life technologies, Darmstadt, Germany). Ports of 384-well-format microfluidic cards were filled with 100 μl sample solution, briefly centrifuged twice for 1,500X g, and sealed. Each 384-well microfluidic card contained the specific TaqMan primers to detect 48 genes (46 target genes and 2 housekeeping genes) for 16 samples per plate. TaqMan primers were selected as the best coverage for the specific genes, as recommended by the manufacturer. For reference gene normalization, eukaryotic 18S rRNA (Assay Hs99999901_s1) and beta-2-microglobulin (B2 M, Rn00560865_m1) were selected. Those reference genes were not found regulated in pilot diabetes studies. In an initial step, the plate was heated to 95 °C (1 °C/s to 50 min, then 50 °C for 2 min, 1 °C/s to 95 °C, then 95 °C for 10 min). Subsequently, a real-time PCR was performed with a maximum of 40 cycles each consisting of sub-step 1 (95 °C for 15 s, rapid cooling to 60 °C with 1 °C/s) and sub-step 2 (60 °C for 1 min, then rapid heating to 95 °C with 1 °C/s). A threshold quantification cycle (C_q_) was evaluated for each gene using the manufacturer’s software. Stability of reference gene expression, sample integrity, and differentially regulated target genes were calculated using the BestKeeper-algorithm, an Excel-based tool using pairwise correlations [9]. Relative target gene expression was finally calculated as 2^−∆C_q_, with ∆C_q_ being the difference between reference median and target gene C_q_ value.

### Statistics

Data were analyzed for normality and for homogeneous variances (Levene). In case of Gaussian distributions, ANOVA was employed. In case of heterogeneous variances and/or non-Gaussian distribution, a Kruskal–Wallis test was used. *P* values <0.05 were regarded as statistically significant. Data are presented as mean ± SEM for gene expression and mean ± SD for capillary and cell counts.

## Results

At 3 months of age, male obese animals had developed stable hyperglycemia (20.7 ± 1.3 mmol/L plasma glucose vs. 6.5 ± 0.1 mmol/L in lean; *P*), which remained on this plateau for the remaining 5 months of observation (Table 1). Hyperinsulinemia initially present in obese rats at 2 months (10.5 ± 0.7 μg/L plasma insulin vs 0.2 ± 0.04 μg/L in lean) decreased due to ß-cell insufficiency at 3 months (3.9 ± 0.6 vs. 0.5 ± 0.09 μg/ml in lean).

**Table 1:** Hyperglycemia, hyperinsulinemia and selected retinal parameters determined in the course of the study

Age	2 months		3 months		8 months	
Biomarker	Lean ZDF	Obese ZDF	Lean ZDF	Obese ZDF	Lean ZDF	*Obese ZDF*
Blood glucose (mmol/L)	6.5 ± 0.1	13.6 ± 1.3	6.8 ± 0.1	20.7 ± 1.3	6.7 ± 0.1	22.4 ± 0.7
Plasma insulin (μg/L)	0.2 ± 0.04	10.5 ± 0.7	0.5 ± 0.09	3.9 ± 0.6	3.4 ± 0.2	3.8 ± 0.2
Number of retinal acellular capillaries (mm^−2^)	n.d.	n.d.	n.d.	n.d.	15 ± 4	24 ± 5
Number of retinal endothelial cells (mm^−2^)	n.d.	n.d	n.d.	n.d.	3,266 ± 415	3,800 ± 438
Number of retinal pericyte cells (mm^−2^)	n.d.	n.d.	n.d.	n.d.	2,238 ± 309	1,618 ± 241

**Fig. 1:**
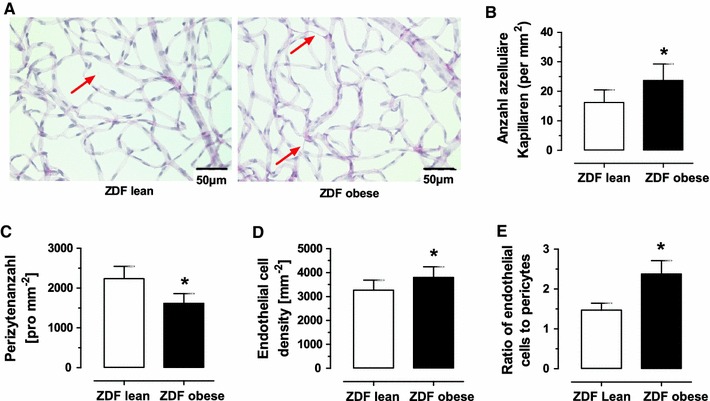
Morphological changes in retina from aged male obese type-2 diabetic ZDF rats. **a** Representative PAS-stained retinas of lean and obese ZDF rats. *Red arrows* point to AC. **b** Quantitative assessment of AC. **c** and **d**. Count in pericytes and endothelial cells. E. Ratio of endothelial cell to pericyte numbers. *Asterisk* denotes *P* < 0.05 (color figure online)

Retinal morphology assessed at 8 months of age revealed an increase in the number of AC in obese (24 ± 5/mm^2^) versus lean ZDF rats (15 ± 4/mm^2^, Fig. 1; Table 1). Pericyte number was reduced by 28 % in obese (1,618 ± 241/mm^2^) versus lean animals (2,238 ± 309/mm^2^), and endothelial cell counts significantly increased by 16 % (3,800 ± 438/mm^2^ in obese vs. 3,266 ± 415/mm^2^ in lean).

**Fig. 2:**
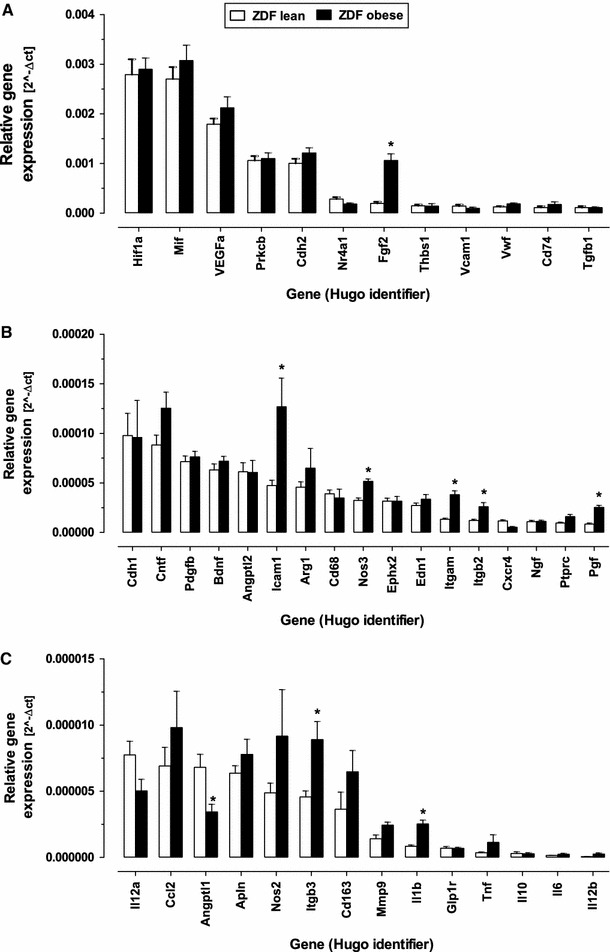
Quantitative assessment of gene expression by microfluidic card PCR. Relative expression to the geometric mean of three housekeeping genes is given and categorized into high (**a**), medium (**b**), and low abundant (**c**) expression. *Asterisk* denotes *P* < 0.05

VEGFa, MIF, and HIF-1α were the most abundantly expressed genes in retinas from both obese and lean ZDF rats, but not differentially regulated (Fig. 2). bFGF (FGF2) and placental growth factor (PGF) were significantly expressed and up-regulated in diabetic animals (5.4X for bFGF and 2.6X for PGF). Specific adhesion molecules, ITGAM, ITGB2, and ICAM1, were found to be significantly up-regulated in the diabetic environment. Nearly all inflammatory genes in our set were either not expressed or expressed at very low abundance. A significant up-regulation on a very low expression level could be observed for Il-1ß, but not for TNFα and IL-6.

## Discussion

Our study shows that in a rat model of type-2 diabetes, retinal vasoregression associated with regulations in specific growth factors and adhesion molecules as reflected by a quantitative gene expression protocol.

Strength of microfluidic card PCR technology is that gene expression of a broader number of genes can be rapidly quantified and compared with very high accuracy. As a limitation, not all known genes can be assessed in an unbiased manner, and hence, the pre-selection of genes to be investigated is of utmost importance. We therefore focused on genes, which have been described so far in several models of diabetic retinopathy and compared quantitatively their relative contribution in type-2-like diabetic retinopathy.

In line with Behl et al. [4], we observed in aged male obese ZDF rats an increased number of acellular retinal capillaries and a pericyte dropout. These morphological changes were similar to changes observed in type-1 diabetes models [10]. Among the investigated genes, only a few are differentially regulated but those may hint to important underlying molecular mechanisms.

Inflammatory genes such as TNFα, interleukin-1ß, and inducible nitric oxide synthase were found to be expressed at a low abundant level, supporting the view of a low-grade inflammatory component. The simultaneous up-regulation of the ITGAM/ITGB2 complex and ICAM-1 provides evidence to an increased leukocyte recruitment process [11]. As noted recently, these entrapped leukocytes may directly damage retinal endothelial cells. However, the overall contribution of leukostasis to retinal disease progression is questionable, since vasoregression can be prevented by different treatment options without significant reduction in leukostasis [12, 13].

High abundant expression was observed for all growth factors investigated, but differential expression between type-2 diabetic and non-diabetic animals could be observed only for bFGF and PGF. Basic fibroblast growth factor (FGF2) is constitutively expressed in the retina, and its expression is increased by a microlesions but the role in preclinical models of retinal vascularization is still uncertain [14]. In clinical diabetic retinopathy, the normal retinal distribution of bFGF changed during the development of diabetic retinopathy and correlated with the onset of retinal neovascularization [15]. bFGF was also involved in repair of laser-induced retinal cell damage, suggesting that bFGF represents, at least in part, a survival factor for damaged neuroglial cells. Activity of PGF in diabetic retinopathy is characterized marginally. Viral-mediated over-expression of PGF in non-diabetic and diabetic rat retinas resulted in a similar up-regulation as observed in our study and contributed to capillary changes and blood–retinal barrier breakdown [16].

Within our selection of genes, VEGF was the growth factor with the highest expression but no significant differential regulation. Others had demonstrated that VEGF mRNA and protein expression is increased in retinae of obese versus lean ZDF rats, albeit at younger disease age and with only a modest up-regulation [6]. In our study, the baseline expression of differentially regulated growth factors such as bFGF and PGF was 550X and 22X higher compared to TNFα. Although this does not rule out any role of low-grade inflammation in the disease progression, it clearly demonstrates that future work should also focus again on specific growth factors, bFGF and PGF, and their contribution as pathological but also survival factors within the retinal neurovascular units.

Even though the insulin level is close to be similar in the diabetic versus non-diabetic rats at 8 months of age, the number of AC and pericyte loss are significantly different as is the blood glucose level. This is in accordance with previous findings that glucose, but not insulin, may determine the biochemistry of microvascular damage (for a review, see [17]). Furthermore, differences in pericyte loss and vasoregression are more likely a consequence of altered glucose-driven pathways than of insulin signaling alterations [18]. It may be interesting to compare retinal vasoregression in ZDF rats versus Zucker fatty rats (ZF rats), which displays persistently high levels of insulin but no hyperglycemia. If vasoregression indeed cannot be identified in ZF rats, this would finally support the role of hyperglycemia as initiating factor.

The finding that not VEGF but bFGF predominates in the diabetic retina at this time point may be indicative of a differential response to injury, combining the tissue response to biochemical stress and the patterning of growth factor expressions in various cells of the retina. Although both VEGF and bFGF are predominantly expressed by Müller cells, the temporal expression may vary. Further studies including earlier time points may be helpful to unravel differences in temporal expression patterns of VEGF, bFGF, and PGF.

In summary, our study identified a strong expression gradient of different genes involved in the pathogenesis of diabetic retinopathy. Most of the inflammatory markers were found at the low end of expression. Given this interesting molecular patterning and reliable morphological changes, we confirmed that male obese ZDF rats are suitable preclinical model for testing new therapeutic approaches in diabetic retinopathy.

## Electronic supplementary material

Below is the link to the electronic supplementary material.

Supplementary material 1 (DOC 95 kb)
