# Supplementary material for: Expression patterning reveals retinal inflammation as a minor factor in experimental retinopathy of ZDF rats
Source: Acta Diabetol. 2014 Jan 30;51(4):553–8. doi: 10.1007/s00592-013-0550-2 (PMC4127441; doi:10.1007/s00592-013-0550-2)
Supplement: Supplementary file 1 — Supplementary material 1 (DOC 95 kb) [file 592_2013_550_MOESM1_ESM.doc]

**Supplement information for gene expression measurements (Wohlfart et al.)**

In this supplement, information is listed to follow the MIQE Guidelines for publication of quantitative real-time PCR Experiments (Bustin *et al*., 2004; Clinical Chemistry 55:4, 611-622).

**Nucleic acid extraction**

Whole rat retinae were used to isolate RNA in two steps. Crude RNA was isolated from individual retina homogenized each in 1 ml Trizol reagent according to the manufacturer’s instructions without modifications (Invitrogen, Karlsruhe, Germany). In a second step, RNA samples were re-purified using RNeasy Mini kits according to the manufacturer’s instructions without modifications (Qiagen, Hilden, Germany) on a semi-automatic QiaCube purification device. This standard purification protocol is listed by the manufacturer as

ProtocolSheet_RNA_RNeasyMini_AnimalTissuesAndCells_DNaseDigest_V2. It included a digestion steps of genomic DNA of the samples bound to the mini columns with RNase-free DNase-I (cat# 79254, Qiagen, Hilden, Germany) as digesting enzyme.

After isolation, nucleic acid quantification and contamination was assessed on a NanoDrop ND-1000 spectrophotometer (peqlab Biotechnologie, Erlangen, Germany). Typical yields were 80-120 ng/µl (in overall 50µl water) with an A260/280 ratio from 1.8 to 2.1. Only RNA with a ratio of >1.9 were taken into the further steps, otherwise the sample was re-purified again (without further genomic DNA digestion).

**Reverse transcription**

A high capacity cDNA Reverse Transcription Kit with RNase Inhibitor (cat # 4374966, Invitrogen, Karlsruhe, Germany) was used for quantitative first-strand synthesis of RNA into cDNA based on the use of random primers according to the manufacturer’s instructions without modifications. Briefly, 1µg RNA (in 10µl) per sample was mixed with a 2X reverse transcription master mix, consisting of 10 volume parts reverse transcription buffer, 4 volume parts 25X dNTPs (100mM), 10 volume parts 10X random primers, 5 volume parts MultiScribe™ Reverse Transcriptase (50 U/μL) and 5 volume parts nuclease-free water. The enzyme reaction was carried out in a Thermocycler (T-Professional, Biometra, Göttingen, Germany) warming first for 10 mins to 25°, followed by 120 min at 37°C.

**qPCR target information**

384-well micro-fluidic card technology was employed for real-time PCR using custom-ordered plates already containing pre-specified Taqman primers in the wells of the plate. Taqman primers (Life Technologies, Darmstadt, Germany) were selected based on the manufacturer’s recommendation (“best coverage”). According to the manufacturer’s guarantee, these assays amplify the intended target at least 10 C_q_ values earlier than the gene with the closest sequence homology. Assays run in a no template control (NTC) reaction do not produce detectable amplification signal (C_q_ >38). All assays provide linear qPCR results over a seven-log range of input template using eight 10-fold dilutions (when C_q_ is plotted against log10 input template quantity, R^2^ value ≥0.98). Every assay detect ≥10 copies of target with a C_q_value that is statistically different (p-value <0.05) than that of the NTC. Every assay exhibits 100% ±10% amplification efficiency when tested in reactions over five orders of magnitude of input template. The specific primer assays are listed in supplementary table-1, including specific information for the underlying genes.

| Gene Symbol | Assay ID | Gene Name | Reference Sequence ID | Amplicon Length | Exon-Boundary |
| --- | --- | --- | --- | --- | --- |
| Fgf2 | Rn00570809_m1 | fibroblast growth factor 2 | NM_019305.2 | 63 | 1-2 |
| Vegfa | Rn01511601_m1 | vascular endothelial growth factor A | NM_031836.2 | 69 | 2-3 |
| Pdgfb | Rn01502596_m1 | platelet-derived growth factor beta polypeptide | NM_031524.1 | 65 | 4-5 |
| Pgf | Rn00585926_m1 | placental growth factor | NM_053595.2 | 60 | 2-3 |
| Angptl1 | Rn01497972_m1 | angiopoietin-like 1 | NM_001109383.1 | 92 | 1-2 |
| Angptl2 | Rn00592058_m1 | angiopoietin-like 2 | NM_133569.1 | 88 | 4-5 |
| Ngf | Rn01533872_m1 | nerve growth factor (beta polypeptide) | XM_227525.5 | 114 | 2-3 |
| Bdnf | Rn02531967_s1 | brain-derived neurotrophic factor | NM_012513.3 | 142 | exon-2 |
| Cntf | Rn00755092_m1 | ciliary neurotrophic factor | NM_013166.1 | 83 | 1-2 |
| Thbs1 | Rn01513693_m1 | thrombospondin 1 | NM_001013062.1 | 83 | single exon |
| Tgfb1 | Rn00572010_m1 | transforming growth factor, beta 1 | NM_021578.2 | 65 | 1-2 |
| Tnf | Rn00562055_m1 | tumor necrosis factor | NM_012675.3 | 82 | 3-4 |
| Ccl2 | Rn00580555_m1 | chemokine (C-C motif) ligand 2 | NM_031530.1 | 95 | 1-2 |
| Mif | Rn00821234_g1 | macrophage migration inhibitory factor | NM_031051.1 | 101 | 1-2 |
| Il6 | Rn01410330_m1 | interleukin 6 | NM_012589.1 | 121 | 3-4 |
| Il12a | Rn00584538_m1 | interleukin 12A | NM_053390.1 | 67 | 2-3 |
| Il12b | Rn00575112_m1 | interleukin 12B | NM_022611.1 | 66 | 3-4 |
| Il10 | Rn00563409_m1 | interleukin 10 | NM_012854.2 | 70 | 4-5 |
| Il1b | Rn00580432_m1 | interleukin 1 beta | NM_031512.2 | 74 | 5-6 |
| Cxcr4 | Rn01483207_m1 | chemokine (C-X-C motif) receptor 4 | NM_022205.3 | 102 | 1-2 |
| Ptprc | Rn00709901_m1 | protein tyrosine phosphatase, receptor type C | NM_001109888.1 | 64 | 24-25 |
| Nr4a1 | Rn01533237_m1 | nuclear receptor subfamily 4 | NM_024388.1 | 64 | 2-3 |
| Itgb3 | Rn00596601_m1 | integrin beta 3 | NM_153720.1 | 66 | n.d. |
| Cdh2 | Rn00580099_m1 | cadherin 2 | NM_031333.1 | 75 | 7-8 |
| Cdh1 | Rn00580109_m1 | cadherin 1 | NM_031334.1 | 105 | 3-4 |
| Icam1 | Rn00564227_m1 | intercellular adhesion molecule 1 | NM_012967.1 | 61 | 1-2 |
| Vcam1 | Rn00563627_m1 | vascular cell adhesion molecule 1 | NM_012889.1 | 90 | 7-8 |
| Hif1a | Rn00577560_m1 | hypoxia-inducible factor 1, alpha subunit | NM_024359.1 | 72 | 4-5 |
| Vwf | Rn01492158_m1 | von Willebrand factor | XM_342759.4 | 57 | 39-40 |
| Itgam | Rn00709342_m1 | integrin, alpha M | NM_012711.1 | 76 | 1-2 |
| Itgb2 | Rn01427948_m1 | integrin, beta 2 | NM_001037780.2 | 142 | 14-15 |
| Cd68 | Rn01495634_g1 | Cd68 molecule | NM_001031638.1 | 62 | 5-6 |
| Cd74 | Rn00565062_m1 | CD74 molecule | NM_013069.2 | 68 | 1-2 |
| Cd163 | Rn01492519_m1 | CD163 molecule | NM_001107887.1 | 61 | 10-11 |
| Ephx2 | Rn00576023_m1 | epoxide hydrolase 2, cytoplasmic | NM_022936.1 | 99 | n.d. |
| Prkcb | Rn00562312_m1 | protein kinase C, beta | NM_012713.3 | 120 | 9-10 |
| Nos2 | Rn00561646_m1 | nitric oxide synthase 2, inducible | NM_012611.3 | 77 | 2-3 |
| Nos3 | Rn02132634_s1 | nitric oxide synthase 3, endothelial cell | NM_021838.2 | 117 | exon 26 |
| Arg1 | Rn00691090_m1 | arginase isoform 1 | NM_017134.2 | 76 | 3-4 |
| Mmp9 | Rn00579162_m1 | matrix metallopeptidase 9 | NM_031055.1 | 72 | 12-13 |
| Edn1 | Rn00561129_m1 | endothelin 1 | NM_012548.2 | 88 | 1-2 |
| Apln | Rn00581093_m1 | apelin | NM_031612.2 | 75 | 1-2 |
| B2M | Rn00560865_m1 | beta-2 microglobulin | NM_012512.2 | 86 | 1-2 |
| 18S | Hs99999901_s1 | eukaryotic ribosomal 18S RNA | HSRRN18S | 187 | single exon |

Supplementary table 1: Detailed information on Taqman qPCR assays used to determine gene expression in whole rat retina lysates

**qPCR protocol information**

After reverse transcription, cDNA samples (0.5-1µg in 50µl), were mixed with 50µl 2X universal mastermix (Life technologies, Darmstadt, Germany, cat#4304437) and filled into the ports of the 384 well micro-fluidic card. The cards was briefly centrifuged twice (1min, 1,500 X g) sealed and loaded into a Viaa-7 cycler. In an initial step, the plate was heated to 95°C (1°C/s to 50 min, then 50°C for 2 min, 1°C/s to 95°C, then 95°C for 10 min). Then, a real time PCR was performed with a maximum of 40 cycles each consisting of sub-step 1 (95°C for 15s, rapid cooling to 60°C with 1°C/s) and sub-step 2 (60°C for 1 min, then rapid heating to 95°C with 1°C/s). FAM fluorescence was continuously monitored. At the end of the PCR, an automatic baseline subtraction was performed using the Viaa-7 software algorithms. For each gene and sample, an automatic threshold quantification cycle (Cq) was calculated and inspected visually to be in the linear phase of a lg (fluorescence) plot (as function of Cq). For reference gene normalization, eukaryotic 18S rRNA (assay id Hs99999901_s1) and beta-2-microglobulin (B2M, assay id Rn00560865_m1) were selected. Those reference genes were not found regulated in pilot diabetes studies. Stability of reference genes expression, sample integrity and differentially regulated target genes were calculated using the BestKeeper – Excel-based tool using pair-wise correlations (Pfaffl *et al*., 2004) Relative target gene expression was finally calculated as 2^-∆Cq with ∆Cq being the difference between reference median and target gene Cq value.
